# Fuzzy Logic and Fuzzy-Based Artificial Intelligence in Dentistry: A Systematic Review

**DOI:** 10.3390/diagnostics16172787

**Published:** 2026-08-30

**Authors:** Martin Baxmann, Márton Zsoldos, Krisztina Kárpáti

**Affiliations:** Department of Orthodontics and Paediatric Dentistry, Faculty of Dentistry, University of Szeged, Tisza Lajos Körút 64–66, H-6720 Szeged, Hungary

**Keywords:** fuzzy logic, dentistry, artificial intelligence, dental diagnosis, treatment planning, fuzzy systems, clinical decision-making, neuro-fuzzy systems

## Abstract

**Background/Objectives:** Fuzzy logic has been increasingly investigated for managing uncertainty in dental diagnosis, risk assessment, image analysis, and clinical decision support. However, the available literature remains fragmented across computational methodologies and clinical applications. This systematic review synthesized the available evidence regarding the diagnostic, predictive, and clinical decision-support performance of fuzzy logic and fuzzy-based artificial intelligence systems in dentistry. **Methods:** A systematic literature search was conducted in PubMed/MEDLINE, Scopus, Web of Science, Embase, the Cochrane Library, and IEEE Xplore in accordance with PRISMA 2020 guidelines. The review protocol was prospectively registered in the Open Science Framework (Registration No. zs3w6). Study selection was performed independently by two reviewers, while data extraction and methodological quality assessments were performed by one reviewer and subsequently verified by a second reviewer. Eligible studies evaluated fuzzy logic or fuzzy-based AI methodologies across dental applications. **Results:** Fifteen studies published between 2005 and 2025 met the inclusion criteria. Applications primarily involved dental disease diagnosis, periodontal risk assessment, oral cancer risk prediction, and radiographic image segmentation. Reported diagnostic accuracy ranged from 82.1% to 100%. However, the evidence base consisted predominantly of proof-of-concept, retrospective, or simulation-based investigations using relatively small datasets, with external validation and prospective clinical evaluation rarely reported. **Conclusions:** Fuzzy logic and fuzzy-based AI systems demonstrate potential for uncertainty-sensitive dental applications involving diagnostic decision support, risk assessment, and radiographic image analysis. However, the current evidence remains exploratory and methodologically heterogeneous, with limited prospective validation and minimal real-world clinical implementation. Larger, externally validated studies are needed before routine clinical adoption can be recommended.

## 1. Introduction

Clinical decision-making in dentistry frequently involves uncertainty. Dentists must often integrate patient-reported symptoms, clinical examinations, radiographic findings, and evolving biological changes when establishing diagnoses and treatment plans [1]. In many cases, disease presentation may be gradual, overlapping, or inconsistently expressed between patients, which can complicate interpretation and clinical decision-making [2]. Variability in clinician experience, incomplete patient information, and subjective interpretation of imaging findings may further contribute to diagnostic inconsistency [1,2,3]. These challenges are particularly relevant in areas such as periodontal disease assessment, oral pathology, radiographic interpretation, and treatment planning [4,5,6].

Artificial intelligence (AI) has increasingly been explored in dentistry to support diagnosis, prediction, image interpretation, and workflow efficiency. Many conventional AI approaches rely on machine learning or deep learning systems that analyze large datasets to identify patterns and generate predictions [7]. Although these methods have demonstrated promising performance in several dental applications, they are often based on rigid binary or probabilistic classifications that may not fully capture the ambiguity commonly encountered in clinical practice [7,8]. In addition, some AI systems function as “black boxes,” meaning that the reasoning behind their outputs may not be easily interpretable by clinicians [9]. Concerns regarding transparency, explainability, and integration into routine clinical workflows continue to represent important barriers to broader clinical adoption [10].

Fuzzy logic represents a computational approach specifically designed to manage uncertainty and approximate reasoning [11]. Unlike conventional binary logic systems, which classify information into fixed categories such as “true” or “false,” fuzzy logic allows information to exist within partial degrees of membership [12]. For example, a clinical finding may be interpreted as partially mild and partially moderate rather than belonging exclusively to a single category. This framework may better reflect real-world clinical reasoning, where disease severity and symptom interpretation often exist along a continuum rather than within sharply defined boundaries [11]. Over time, several fuzzy-based methodologies have been developed, including fuzzy inference systems, fuzzy expert systems, neuro-fuzzy systems, fuzzy clustering techniques, hybrid fuzzy-AI models, and intuitionistic fuzzy systems [13]. Although these methodologies differ in their computational implementation, they share the common feature of using fuzzy logic as the primary framework for representing uncertainty and supporting inference or decision-making.

Within dentistry, fuzzy methodologies have primarily been investigated in applications involving diagnostic support systems, periodontal disease assessment, oral cancer risk prediction, and dental image analysis [14]. These systems have been explored as standalone computational models and components of hybrid AI frameworks intended to support uncertainty-aware clinical reasoning and radiographic interpretation [14]. In imaging applications, fuzzy clustering approaches have also been investigated for improving segmentation and interpretation of dental radiographs and panoramic X-ray images, particularly in environments involving low contrast, noise, or overlapping anatomical structures [15].

Despite increasing interest in fuzzy methodologies within dental research, the existing literature remains fragmented across different computational approaches, study designs, and clinical applications. Considerable variation exists in model architecture, validation strategies, datasets, outcome reporting, and methodological quality, making it difficult to evaluate the broader applicability and translational potential of these systems within dental practice. Furthermore, many published investigations are exploratory or proof-of-concept studies with limited external validation and uncertain clinical generalizability. To date, no comprehensive systematic review has synthesized the available evidence regarding fuzzy logic and fuzzy-based AI systems in dentistry.

Therefore, the aim of this systematic review was to synthesize the available evidence regarding the diagnostic, predictive, and clinical decision-support performance of fuzzy logic and fuzzy-based artificial intelligence systems in dentistry, together with their methodological characteristics and potential clinical utility. The review question was developed according to the PICOS framework and asked: What evidence supports the diagnostic, predictive, and clinical decision-support performance of fuzzy logic and fuzzy-based artificial intelligence systems in dentistry?

## 2. Materials and Methods

This systematic review was conducted using established systematic review methodology and is reported in accordance with the Preferred Reporting Items for Systematic Reviews and Meta-Analyses (PRISMA 2020) Statement (Supplementary Table S1). The review protocol was prospectively registered in the Open Science Framework (OSF; Registration No. zs3w6), which provides a publicly accessible, time-stamped record of the review protocol and any protocol refinements made before study execution. Minor modifications to the registered protocol were made before study execution in response to the limited quantity and methodological diversity of the available literature. Specifically, the original protocol focused on fuzzy logic–based methodologies for dental diagnosis and decision support. During protocol refinement, additional contemporary fuzzy-based methodologies (e.g., neuro-fuzzy systems, hybrid fuzzy models, and advanced clustering approaches) were explicitly incorporated into the predefined data extraction framework because they represented methodological variations of the same underlying computational approach rather than new review domains.

The review question was developed according to the PICOS framework and aimed to synthesize the available evidence regarding the diagnostic, predictive, and clinical decision-support performance of fuzzy logic and fuzzy-based artificial intelligence systems in dentistry. The population comprised dental patients or dental datasets, the intervention consisted of fuzzy logic or fuzzy-based artificial intelligence methodologies, comparators included conventional diagnostic approaches or alternative computational methods where applicable, and the primary outcomes were diagnostic, predictive, and clinical decision-support performance. Studies were eligible for inclusion if they evaluated fuzzy logic or fuzzy-based AI methodologies within dentistry, including fuzzy inference systems, fuzzy expert systems, neuro-fuzzy systems, intuitionistic fuzzy systems, fuzzy clustering approaches, hybrid fuzzy-AI models, or related fuzzy methodologies. For the purposes of this review, fuzzy-based artificial intelligence was defined as computational systems in which fuzzy logic constituted the primary decision-making or inference methodology, either as a standalone approach or integrated within hybrid architectures such as neuro-fuzzy or fuzzy clustering systems. Hybrid models were eligible only when fuzzy logic remained a core component of the computational framework. Eligible applications included dental diagnosis, prediction, treatment planning, image analysis, classification, risk assessment, and clinical decision support. Studies reporting diagnostic, predictive, computational, methodological, or clinical outcomes were considered eligible. Included study designs comprised randomized controlled trials, cohort studies, case-control studies, cross-sectional studies, observational studies, diagnostic accuracy studies, retrospective modeling studies, and methodological investigations available as full-text publications. Given the limited and emerging evidence base in this field, eligible full-text primary research was not restricted to peer-reviewed journal articles; full-text conference proceedings and preprints were also considered when they provided sufficient methodological and outcome data for eligibility assessment and evidence synthesis. Systematic reviews and meta-analyses were excluded to avoid duplication of evidence and ensure that the synthesis was based exclusively on primary studies.

Studies were excluded if they were unrelated to dentistry, evaluated AI systems without fuzzy logic–based methodologies, or lacked sufficient methodological detail or outcome reporting. Editorials, narrative reviews, systematic reviews, meta-analyses, opinion articles, conference abstracts without accessible full text, book chapters, and other non-research publications were excluded.

A comprehensive literature search was performed across PubMed/MEDLINE, Scopus, Web of Science, Embase, Cochrane Library, and IEEE Xplore. Search strategies were developed using combinations of controlled vocabulary terms and free-text keywords related to dentistry and fuzzy methodologies and were adapted for each database according to its indexing system, search syntax, and functionality. Database-specific adaptations included modification of field tags, controlled vocabulary, Boolean syntax, truncation, and proximity operators where supported by the individual platform. For engineering-oriented databases such as IEEE Xplore and Scopus, equivalent free-text terms were mapped to the database-specific searchable fields and syntax while preserving the core concepts of dentistry and fuzzy methodologies. No restrictions on publication date or language were applied. Manual reference screening and citation tracking were additionally performed to identify potentially relevant studies not retrieved through electronic database searches.

The PubMed search strategy was as follows: ((“dentistry”[Title/Abstract] OR “dental”[Title/Abstract] OR “oral”[Title/Abstract]) AND (“fuzzy”[Title/Abstract] OR “fuzzy logic”[Title/Abstract] OR “fuzzy systems”[Title/Abstract] OR “fuzzy inference”[Title/Abstract] OR “fuzzy expert system*”[Title/Abstract] OR “neuro-fuzzy”[Title/Abstract] OR “intuitionistic fuzzy”[Title/Abstract])).

All retrieved records were imported into Covidence systematic review software for deduplication, screening, and study selection management [16]. Title and abstract screening, followed by full-text assessment of potentially eligible studies, was performed independently by two reviewers according to predefined eligibility criteria. Each retrieved record was assessed by both reviewers, and any disagreements regarding study eligibility were resolved through discussion and consensus.

Data extraction was performed by one reviewer using a standardized spreadsheet designed to capture study characteristics, dental application areas, fuzzy methodology types, model purposes, dataset characteristics, validation approaches, comparator methods, outcome measures, key findings, and reported limitations. All extracted data were subsequently checked against the original studies by a second reviewer, and any discrepancies were resolved through discussion and consensus. Extracted variables included study design, dental specialty area, fuzzy system architecture, dataset size, validation methods, diagnostic or computational performance metrics, and clinical applicability.

The methodological quality and risk of bias of included studies were evaluated using appraisal tools appropriate to study design. Diagnostic accuracy studies were assessed using QUADAS-2, prediction modeling studies were evaluated using PROBAST where applicable, and observational studies were assessed using Joanna Briggs Institute (JBI) critical appraisal tools. For primarily methodological, computational, or heuristic rule-based studies that did not conform fully to conventional clinical prediction-model designs, appraisal criteria were interpreted in the context of the study design, with emphasis on methodological transparency, dataset reporting, reproducibility, validation procedures, reference standards or expert comparators where applicable, and completeness of outcome reporting. Accordingly, PROBAST-based judgments for heuristic expert systems were interpreted as indicators of limitations in prediction-model validation rather than as direct evidence that the underlying computational approach was methodologically invalid. Risk-of-bias assessments were conducted by two reviewers using the predefined appraisal tools. The assessment workload was divided between the reviewers according to study design, with each study initially assessed by one reviewer. All assessments were subsequently checked and verified by the other reviewer, and any discrepancies or uncertainties regarding methodological quality or risk-of-bias judgments were resolved through discussion and consensus.

Given the variation in study designs, clinical applications, and reported outcomes, findings were synthesized using a structured narrative approach. Included investigations were grouped according to dental application area, fuzzy methodology type, and primary clinical objective. The synthesis focused on fuzzy system architectures, validation strategies, datasets, performance outcomes, interpretability, and clinical applicability. Quantitative synthesis and meta-analysis were initially planned for sufficiently homogeneous diagnostic and classification outcomes. However, meta-analysis was ultimately not performed because the included studies were too heterogeneous with respect to study methodology, fuzzy system architectures, datasets, validation procedures, comparator methods, and reported outcome measures.

## 3. Results

### 3.1. Study Selection

**Figure 1:**
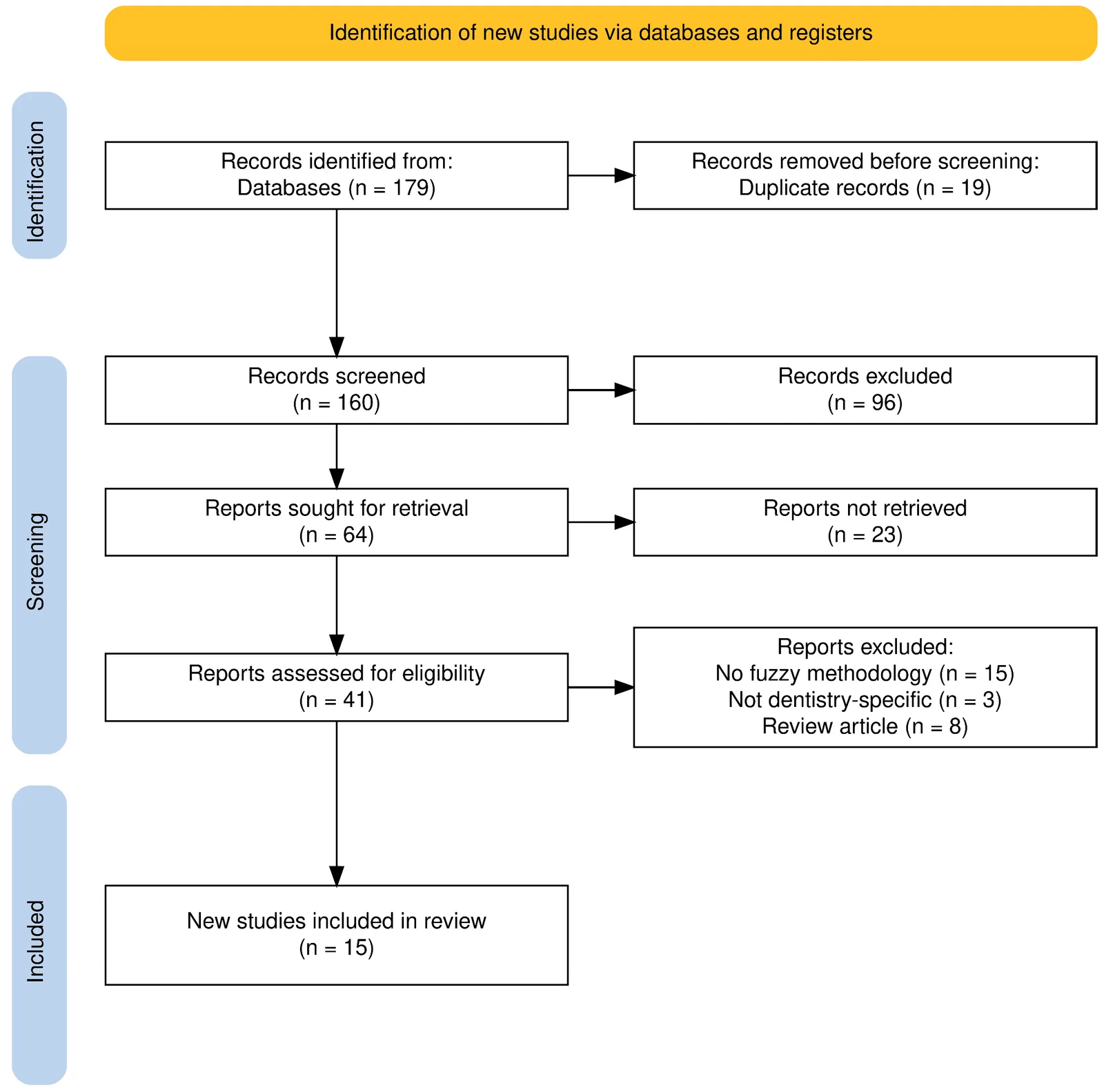
PRISMA flow diagram.

The electronic database searches identified 179 records. After removal of 19 duplicate records, 160 studies remained for title and abstract screening. Of these, 96 records were excluded, resulting in 64 reports sought for retrieval. Twenty-three reports could not be retrieved, leaving 41 reports assessed for eligibility. Following full-text review, 26 studies were excluded for not meeting the inclusion criteria, including studies that did not utilize fuzzy methodology (*n* = 15), were not specific to dentistry (*n* = 3), or were review articles (systematic reviews, meta-analyses, or narrative reviews) (*n* = 8) (Supplementary Table S2). Ultimately, 15 studies met the eligibility criteria and were included in the final review (Figure 1).

### 3.2. Study Characteristics

The 15 included studies were published between 2005 and 2025 and originated primarily from Asia, Europe, and the Middle East. Most investigations were observational, methodological, or proof-of-concept studies evaluating fuzzy logic-based systems for diagnostic support, risk assessment, radiographic analysis, or broader clinical decision-support applications within dentistry. The included studies varied widely in study design, dataset size, computational architecture, validation methodology, and outcome reporting. Several studies relied on prototype or simulation-based datasets, while others incorporated clinical patient cohorts or larger radiographic image datasets for algorithm development and testing. A wide range of fuzzy methodologies was identified across the included literature, including Mamdani fuzzy inference systems, fuzzy expert systems, multilayer fuzzy inference systems, neuro-fuzzy models, fuzzy clustering techniques, Gaussian kernel-based conditional spatial fuzzy clustering, neutrosophic-fuzzy hybrid systems, and certainty factor–integrated fuzzy frameworks [17,18,19,20,21]. Validation approaches included expert comparison, confusion matrix analysis, segmentation performance metrics, statistical correlation analyses, and prototype system testing [22,23,24]. A summary of the characteristics of the included studies can be found in Supplementary Table S3.

### 3.3. Clinical Diagnostic and Risk Assessment Applications

Most included studies focused on fuzzy logic-based systems for dental disease diagnosis, periodontal disease assessment, oral cancer risk assessment, or clinical decision-support applications. Symptom-based diagnostic models commonly incorporated clinical variables such as plaque accumulation, gingival inflammation, pain severity, bleeding, halitosis, gum swelling, probing pocket depth, attachment loss, and tooth mobility to classify conditions including gingivitis, periodontitis, pulpitis, and other oral diseases [18,20,25,26,27]. Most systems were constructed using predefined linguistic variables and expert-derived IF–THEN rule structures based on clinical symptom severity and diagnostic thresholds [21,25].

Several investigations incorporated hybrid or multilayer fuzzy frameworks for classification and decision-support applications [20,28]. Multilayer fuzzy inference systems combined with K-means clustering were applied for classification of pulpitis, gingivitis, periodontitis, and advanced periodontitis using symptom severity scoring and centroid-based membership functions [20]. Certainty factor integration was additionally used to improve diagnostic weighting and disease classification in fuzzy expert systems for dental and oral diseases [18]. Neuro-fuzzy and coactive neuro-fuzzy architectures were also identified within systems designed for adaptive diagnostic reasoning and clinical support [21].

Risk assessment applications represented an additional subgroup of studies. Fuzzy multicriteria decision-support systems were developed for oral cancer risk estimation using oxidative stress biomarkers including malondialdehyde and proton donor capacity [29]. AI-based fuzzy logic systems were also developed for estimating periodontitis risk in patients with type 2 diabetes mellitus using variables such as body mass index, glycemia, triglycerides, and cholesterol levels [23]. Additional applications included fuzzy-assisted assessment of dental care safety during the COVID-19 pandemic and fuzzy classification systems for evaluating patient satisfaction and quality of primary dental care services [30,31].

### 3.4. Imaging and Segmentation Applications

A substantial subgroup of studies focused on radiographic image analysis and segmentation using fuzzy clustering and hybrid fuzzy methodologies. These investigations primarily involved panoramic dental radiographs and digital dental X-ray datasets for applications including tooth component segmentation, jaw lesion identification, image enhancement, and structural classification of enamel, dentine, pulp, and surrounding tissues [17,19,22]. These studies specifically addressed challenges associated with low image contrast, grayscale overlap, image noise, and unclear anatomical boundaries within dental radiographs.

Fuzzy C-means clustering represented the most frequently used methodology within imaging applications. Modified clustering frameworks incorporating spatial information, Gaussian kernel distances, gradient orientation mapping, or neutrosophic approaches were developed to improve segmentation accuracy and reduce sensitivity to image noise and outliers [17,19,22]. Gaussian kernel-based conditional spatial fuzzy C-means algorithms were used for segmentation of dental structures including enamel, dentine, pulp, and background regions in panoramic radiographs, whereas gradient orientation mapping–based fuzzy clustering models incorporated structural and spatial image features for digital dental X-ray segmentation [19,22]. Hybrid neutrosophic-fuzzy clustering techniques were additionally applied for automatic jaw lesion segmentation and lesion boundary detection in panoramic imaging datasets [17]. These imaging and segmentation studies generally reported quantitative performance metrics including sensitivity, specificity, precision, similarity coefficients, true-positive rates, and overall segmentation accuracy [17,19,22]. Most investigations evaluated retrospective radiographic datasets or computational prototype performance.

### 3.5. Performance Outcomes

Most included studies reported favorable diagnostic, classification, or segmentation outcomes for fuzzy logic–based systems. Among clinical diagnostic investigations, reported classification accuracy generally ranged from 82.1% to 100% depending on the dataset, methodology, and validation approach used [18,20,24,27]. Diagnostic systems integrating certainty factor methodologies with fuzzy logic reported overall diagnostic accuracy values exceeding 94% for dental and oral disease classification [18]. Comparative evaluation between Mamdani fuzzy logic and Naïve Bayes classification approaches demonstrated slightly higher reported accuracy for fuzzy logic systems in dental disease detection [24].

Studies involving periodontal and oral cancer risk assessment primarily reported successful risk stratification and numerical classification outputs rather than conventional diagnostic accuracy metrics. Fuzzy multicriteria decision-support systems generated graded oral cancer risk estimations using oxidative stress biomarker profiles, whereas fuzzy logic systems for periodontitis risk assessment demonstrated statistically significant relationships between cumulative fuzzy risk scores and clinical periodontal parameters in diabetic patient cohorts [23,29]. Imaging and segmentation investigations also reported generally favorable quantitative performance metrics. Segmentation accuracy values approaching or exceeding 95% were reported in studies involving dental radiographs and panoramic X-ray datasets [22]. High sensitivity, specificity, precision, and similarity coefficients were additionally observed in studies evaluating hybrid fuzzy clustering and neutrosophic segmentation approaches for jaw lesion identification and tooth component segmentation [17,19].

### 3.6. Risk of Bias and Methodological Quality

Overall, the risk-of-bias assessment identified substantial methodological limitations across the included studies (Supplementary Table S4). Most investigations relied on relatively small datasets, retrospective or simulation-based validation procedures, prototype systems, or limited comparator analyses. External multicenter validation and prospective clinical implementation were infrequently reported across both diagnostic and imaging studies. Several investigations additionally lacked detailed reporting regarding dataset selection, model calibration, reproducibility procedures, or independent testing cohorts.

Among diagnostic and decision-support studies, many systems were evaluated using expert comparison or internally derived datasets without external validation, limiting assessment of generalizability and real-world clinical performance [18,21,27]. Several studies also relied on manually defined fuzzy membership functions and expert-derived rule structures without standardized optimization or reproducibility assessment [25,26]. Imaging investigations generally reported more quantitative validation metrics; however, most studies remained computationally focused and lacked prospective radiographic validation or integration into routine clinical imaging workflows [17,19,22]. Differences in methodology, outcome reporting, validation strategies, and dataset composition limited direct comparisons across studies and prevented meaningful quantitative synthesis.

## 4. Discussion

This systematic review evaluated the current evidence regarding the application of fuzzy logic and fuzzy-based AI systems in dentistry. Across the included literature, fuzzy methodologies were most commonly applied to dental disease diagnosis, periodontal disease assessment, oral cancer risk assessment, clinical decision support, and radiographic image segmentation. Despite marked differences in study design, computational architecture, dataset composition, and validation methodology, several consistent trends emerged across the reviewed studies. Clinical diagnostic applications were predominantly based on rule-driven fuzzy inference systems using symptom-based inputs, whereas imaging applications more frequently utilized fuzzy clustering approaches and hybrid segmentation frameworks for panoramic radiographs and dental X-ray analysis [17,22,25,27]. More recent investigations additionally incorporated multilayer architectures, certainty factor integration, and neuro-fuzzy systems intended to improve diagnostic classification and uncertainty handling within increasingly complex computational frameworks [18,20,21]. Despite searching six major electronic databases, only 15 studies published between 2005 and 2025 met the eligibility criteria. This modest evidence base suggests that fuzzy logic remains a relatively specialized area of computational dental research rather than an established component of routine clinical investigation. Although the limited number of eligible studies may partly reflect the stringent inclusion criteria and requirement for sufficient methodological and outcome reporting, it also highlights the need for more robust primary research evaluating fuzzy-based methodologies in clinically relevant settings.

Most included studies reported favorable diagnostic or segmentation performance outcomes, with several investigations demonstrating high reported accuracy, sensitivity, specificity, and segmentation metrics under controlled experimental conditions [18,19,22,24]. Imaging studies generally incorporated more quantitative performance evaluation methods, including similarity coefficients, true-positive rates, and segmentation accuracy measures, whereas clinical diagnostic investigations more commonly relied on expert comparison or classification accuracy reporting [17,19,22,24,27]. Studies of oral cancer and periodontitis risk assessment also demonstrated the ability to generate graded risk classifications using biomarker-based fuzzy inference systems [23,29]. Despite these generally favorable performance metrics, interpretation should remain cautious. Many studies relied on small datasets, retrospective analyses, simulation-based validation, or prototype systems, and relatively few evaluated clinical implementation or external validation. Consequently, high reported diagnostic accuracy may not necessarily translate into reliable performance under routine clinical conditions.

Methodological standardization was also limited, particularly in fuzzy system construction, membership function definition, dataset selection, validation strategy, and outcome reporting, reducing comparability and reproducibility across studies. In several investigations, reproducibility assessment and independent external testing cohorts were either incompletely reported or absent altogether. Furthermore, although many systems demonstrated strong computational performance within isolated experimental settings, relatively few studies evaluated integration into real-world dental workflows or assessed usability within routine clinical practice environments. To facilitate comparison across the diverse applications and study designs, Table 1 provides a structured synthesis of the major application domains, fuzzy methodologies, reported outcomes, and recurring limitations.

The reviewed literature nevertheless suggests continued interest in fuzzy logic methodologies for uncertainty-sensitive dental applications involving partially overlapping clinical findings and imprecise diagnostic boundaries. Several studies specifically utilized linguistic variables and membership functions to model symptom severity ranges and graded clinical presentations that may be difficult to classify using rigid binary approaches [18,25,29]. However, while these systems demonstrate potential applicability for uncertainty-aware decision support and radiographic analysis, the currently available evidence remains insufficient to support routine clinical implementation or broader clinical adoption.

This review has several limitations that should be acknowledged. First, the included studies varied considerably in study design, computational architecture, validation procedures, and reported outcomes, limiting the comparability of findings across investigations. Second, a large proportion of the included literature consisted of engineering-oriented or prototype computational studies with limited clinical implementation. Third, inconsistent reporting of datasets, validation methods, and comparator systems reduced reproducibility assessment and limited direct comparison across investigations. Fourth, because of the relatively small number of eligible studies and the heterogeneity of reported outcomes, quantitative synthesis and meta-analysis were not feasible. In addition, data extraction was performed by one reviewer and subsequently checked against the original studies by a second reviewer rather than being performed independently in duplicate. Although second-reviewer verification was used to identify and resolve discrepancies, this approach may have increased the possibility of extraction error compared with fully independent duplicate extraction. Finally, 23 reports could not be retrieved for full-text assessment despite comprehensive database searching. The inability to assess these reports may have introduced retrieval or selection bias if the unavailable studies differed systematically from those that were successfully retrieved.

Future research should prioritize prospective multicenter studies using larger and more diverse patient populations, external validation, and standardized reporting methodologies. Studies should also evaluate reproducibility, clinical usability, workflow integration, and implementation feasibility to determine whether favorable computational performance translates into meaningful improvements in clinical decision-making. Direct comparisons with contemporary machine learning and deep learning approaches would further clarify the relative performance and potential clinical utility of fuzzy logic–based AI systems in dentistry.

## 5. Conclusions

The available evidence suggests that fuzzy logic and fuzzy-based artificial intelligence systems have potential utility for uncertainty-sensitive dental applications involving diagnostic decision support, risk assessment, and radiographic image analysis. However, the current evidence remains limited by methodological heterogeneity, small datasets, limited external validation, and minimal real-world clinical implementation. Prospective multicenter validation studies with standardized reporting and evaluation of clinical workflow integration are needed before these methodologies can be recommended for routine clinical practice.

## Figures and Tables

**Table 1:** Summary of major findings across fuzzy logic applications in dentistry.

Application Domain	Common Fuzzy Methodologies	Common Inputs/Data	Main Reported Outcomes	Recurring Limitations
Dental disease diagnosis [18,24,25,27]	Mamdani fuzzy inference systems, fuzzy expert systems, certainty factor–integrated fuzzy systems, neuro-fuzzy models	Plaque, pain, gingival inflammation, bleeding, halitosis, tooth mobility	Reported diagnostic accuracy generally ranged from 82.1% to 100%	Small datasets, retrospective validation, expert-derived rules, limited external validation
Periodontal and gum disease assessment [23,25,26]	Fuzzy expert systems, AI-based fuzzy risk systems, multilayer fuzzy inference systems	Periodontal indices, probing depth, attachment loss, biomarkers, glycemia, cholesterol, triglycerides	Successful disease classification and graded periodontal risk stratification	Prototype-based systems, limited prospective clinical evaluation
Oral cancer risk prediction [29]	Fuzzy multicriteria decision-support systems	Oxidative stress biomarkers, malondialdehyde, proton donor capacity	Numerical oral cancer risk estimation and successful risk classification	Small patient cohorts, limited biomarker validation
Dental imaging and radiographic segmentation [17,19,22]	Fuzzy C-means clustering, Gaussian kernel–based clustering, neutrosophic-fuzzy hybrid systems	Panoramic radiographs, dental X-rays, grayscale intensity distributions	High reported segmentation accuracy, sensitivity, specificity, and similarity coefficients	Primarily computational validation, lack of clinical workflow integration
Clinical decision support and healthcare assessment [28,30,31]	Fuzzy classification systems	Clinical datasets, patient-reported measures, environmental and safety variables	Successful classification and uncertainty-sensitive decision support	Minimal real-world implementation evaluation

## Data Availability

No new data was created or analyzed in this study. Data sharing is not applicable to this article.

## Supplementary Materials

The following supporting information can be downloaded at https://www.mdpi.com/article/10.3390/diagnostics16172787/s1, Supplementary Table S1: PRISMA checklist; Table S2: Summary of articles excluded at the full-text screening stage; Table S3: Summary of studies included in the review; Table S4: Summary of the risk of bias outcomes.

## Abbreviations

The following abbreviations are used in this manuscript:

AI	Artificial Intelligence
ANFIS	Adaptive Neuro-Fuzzy Inference System
AUC	Area Under the Curve
CANFES	Coactive Neuro-Fuzzy Expert System
CDSS	Clinical Decision Support System
FCM	Fuzzy C-Means
FES	Fuzzy Expert System
FIS	Fuzzy Inference System
GK-csFCM	Gaussian Kernel-Based Conditional Spatial Fuzzy C-Means
OSF	Open Science Framework
PRISMA	Preferred Reporting Items for Systematic Reviews and Meta-Analyses
PROBAST	Prediction Model Risk of Bias Assessment Tool
