# Supplementary material for: Fuzzy Logic and Fuzzy-Based Artificial Intelligence in Dentistry: A Systematic Review
Source: Diagnostics (Basel). 2026 Aug 30;16(17):2787. doi: 10.3390/diagnostics16172787 (PMC13565610; doi:10.3390/diagnostics16172787)
Supplement: Supplementary file 1 [file diagnostics-16-02787-s001.zip › Supplementary Table S2.pdf]

Supplementary Table S2. Summary of articles excluded at the full-text screening stage

Study	Reason for Exclusion
Bakhsh et al. (2025)	No fuzzy methodology
Carvalho et al. (2023)	No fuzzy methodology
Dawa et al. (2025)	No fuzzy methodology
Dziubenko et al. (2025)	No fuzzy methodology
Ezhov et al. (2021)	No fuzzy methodology
Giannakopoulos et al. (2023)	No fuzzy methodology
Glick et al. (2022)	No fuzzy methodology
Hernández-Julio et al. (2019)	Not dentistry-specific
Ignatov et al. (2024)	No fuzzy methodology
Kwiatek et al. (2025)	No fuzzy methodology
Lyakhov et al. (2022)	No fuzzy methodology
Orhan et al. (2023)	No fuzzy methodology
Thanathornwong et al. (2023)	No fuzzy methodology
Turosz et al. (2024)	No fuzzy methodology
Zhang et al. (2024)	No fuzzy methodology
Zhu et al. (2023)	No fuzzy methodology
Chen et al. (2021)	Not dentistry-specific
Navin & Krishnan (2024)	Not dentistry-specific
Aloufi et al. (2022)	Review article
Boreak (2020)	Review article
Das (2025)	Review article
Feher et al. (2024)	Review article
Hung et al. (2022)	Review article
Ibraheem (2024)	Review article
Ismail et al. (2025)	Review article
Kennet & Falkner (2025)	Review article
