# Supplementary material for: Fuzzy Logic and Fuzzy-Based Artificial Intelligence in Dentistry: A Systematic Review
Source: Diagnostics (Basel). 2026 Aug 30;16(17):2787. doi: 10.3390/diagnostics16172787 (PMC13565610; doi:10.3390/diagnostics16172787)
Supplement: Supplementary file 1 [file diagnostics-16-02787-s001.zip › Supplementary Table S3.pdf]

Supplementary Table S3. Summary of studies included in the review

Study	Year	Study design / type	Dental application area	Fuzzy system category	Specific fuzzy/AI method	Purpose/objective	Inputs / data source	Sample / dataset	Comparator / reference standard	Validation method	Performance metrics / findings	Main conclusion / clinical implication
Ambara, Putra, & Rusjayanthi	2017	Expert system development and verification study	Dental and oral disease diagnosis	Fuzzy expert system + certainty factor	Fuzzy logic for user trust/uncertainty values; certainty factor for diagnostic conclusion	Develop an expert system to diagnose dental and oral diseases from user responses/symptoms	27 symptom questions; crisp yes/no inputs and fuzzy certainty inputs	10 verification cases; knowledge base with 27 symptoms and 9 diseases	Dentist/expert diagnosis	Expert-user/system comparison across test cases	Accuracy 94.627%; average difference 5.37%	Fuzzy logic handled symptom uncertainty while certainty factor produced diagnostic output; proposed as an accessible dental/oral disease diagnostic tool
Adwibowo	2020	Fuzzy inference system development; preprint	COVID-19 dental care safety/risk assessment	Fuzzy risk assessment system	Fuzzy inference with fuzzification, rule evaluation, defuzzification	Assess dental care safety/risk based on patient and environmental COVID-19 factors	Patient body temperature, travel history, dental care ventilation rate, disinfection frequency	No clinical patient validation reported; model/simulation study	No formal comparator	Scenario/system output assessment	System identified dental care risk/safety according to patient health status and hygiene/environmental conditions	Fuzzy system proposed for early safety/risk assessment in dental care settings under uncertainty
Allahverdi & Akcan	2011	Conference paper; expert system design	Periodontal dental disease diagnosis and treatment support	Fuzzy expert system	Fuzzy inference/rule-based expert system	Design a fuzzy expert system for diagnosis of periodontal dental disease	Periodontal signs/symptoms and expert-derived variables	No clinical patient sample reported; system development used five clinical/radiographic input variables and 164 expert-derived fuzzy rules, with illustrative patient examples.	Expert knowledge/rules	System demonstration with comparison of FES outputs against MATLAB-generated outputs across 13 input scenarios.	FES and MATLAB outputs were reported as similar across 13 input scenarios; illustrative cases demonstrated classification of gingivitis and severe periodontitis with	Shows early application of fuzzy expert systems to periodontal diagnosis and treatment planning

											corresponding treatment recommendations. No numerical diagnostic accuracy metric was reported.	
Alsmadi	2018	Image segmentation algorithm evaluation	Jaw lesion segmentation on panoramic X-rays	Hybrid fuzzy clustering	Hybrid Fuzzy C-Means + neutrosophic approach	Develop fully automatic segmentation method for jaw lesion regions in panoramic X-ray images	Panoramic jaw X-ray images	95 panoramic jaw X-ray images (60 malignant and 35 benign cases), with diagnoses confirmed by pathology	Manual delineation by oral pathologist; comparison with HFA-FCM and ABC-FCM	Segmentation performance evaluation using area error metrics, sensitivity, specificity, similarity	FP rate 6.1%; TP rate 90%; specificity 0.9412; sensitivity 0.9592; similarity 0.9471	Hybrid FCM/neutrosophic method produced lesion regions most similar to oral pathologist delineation and outperformed comparison algorithms
Fariza, Arifin, Astuti, & Kurita	2019	Image segmentation algorithm evaluation	Tooth component segmentation in dental X-ray images	Fuzzy clustering / spatial FCM	Gaussian kernel-based conditional spatial Fuzzy C-Means (GK-csFCM)	Segment tooth into enamel, dentine, pulp, and background for forensic/dental image analysis	Dental X-ray images preprocessed with CLAHE and gamma adjustment	12 real dental X-ray images of molars selected from panoramic radiographs, with ground-truth tooth-component boundaries verified by a dental forensic expert	Compared with typical FCM and csFCM	Qualitative and quantitative segmentation metrics	GK-csFCM outperformed FCM and csFCM in accuracy, specificity, sensitivity, and precision	Modified spatial FCM improved segmentation under noise, low contrast, and uneven illumination
Buhari & KotherMohideen	2020	Image segmentation algorithm evaluation	Digital dental X-ray image segmentation	Fuzzy clustering / feature-enhanced FCM	Gradient Orientation Mapping Based Fuzzy C-Means (GOMB-FCM)	Improve automated clustering/segmentation of digital dental X-ray images	Dental X-ray images; gradient, RGB, edge intensity, entropy, WLD, texture features	500 dental image datasets; real datasets from dental hospitals 2017–2019; also reports 200 testing images in feature section	Compared with Otsu, FCM, EA-FCM, FCM-SC, and related FCM methods	Cluster validity indices and segmentation comparisons	Proposed method reported improved cluster validity/segmentation performance and GUI implementation in MATLAB 2018	GOMB-FCM integrates spatial/structural/gradient features for improved dental X-ray segmentation

Ismayilova & Tarverdiyeva	2023	Expert system / fuzzy logic study	Dental disease diagnosis	Fuzzy expert system / fuzzy C-means rule extraction	Fuzzy C-Means + fuzzy logic toolbox	Use observed symptom data to distinguish dental diseases and generate diagnoses	8 symptomatic attributes selected through feature selection	100 patient data instances reported in the abstract and conclusion; Methods additionally describe a dataset of 80 diagnosed cases	No independent diagnostic reference standard reported; model inputs and diagnostic rules were informed by observations and expert interviews	Methodological testing of fuzzy C-means/fuzzy toolbox	Authors reported successful identification of dental disease and provided illustrative rule-based classifications; no quantitative diagnostic performance metric was reported	Shows fuzzy C-means can extract rules and support dental disease diagnosis
Nasir et al.	2024	Fuzzy expert system development	Gum disease identification	Fuzzy expert system	MATLAB-based FES with fuzzy rules and defuzzification	Identify gum disease, including gingivitis and periodontitis, using fuzzy expert system	Gum bleeding frequency, plaque accumulation, gum recession, tooth mobility and related clinical variables	Series of constructed gum-disease test cases, with outputs additionally cross-referenced against clinical data from actual patient cases; number of test cases/patient cases not reported by the authors	Expected diagnostic outcomes based on consultation with dental experts and relevant medical literature; system outputs were also cross-referenced with clinical data from actual patient cases	Scenario-based system testing with comparison against expert- and literature-informed expected outcomes and cross-referencing with clinical data from actual patient cases	Illustrative test case produced a fuzzy output value of 0.133, corresponding to a diagnosis of gingivitis; no quantitative diagnostic performance metrics (e.g., accuracy, sensitivity, or specificity) were reported	FES offers nuanced diagnostic support for gum disease and may aid treatment planning

Parewe & Mahmudy	2016	Fuzzy inference system / expert system development	Dental disease identification	Fuzzy inference system	Fuzzy logic with linguistic variables, membership functions, rule base, fuzzification, inference, defuzzification	Identify dental disease based on symptom criteria	8 symptom inputs: plaque, inflamed gums, pain, red gums, swollen gums, bleeding, bad breath, loose teeth; each 0–90 with mild/moderate/severe linguistic terms	20 test cases comprising dental symptom data and corresponding disease classifications	Expert dental judgment used as the reference standard for comparison with system-generated diagnoses	Accuracy testing across 20 cases by comparing FIS-generated diagnoses with expert dental judgments	Diagnostic accuracy of 85% across 20 test cases compared with expert judgment; error rate, 15%	Provides early FIS model for dental disease identification using common periodontal/dental symptoms
Prandana, Mawengkang, & Suwilo	2024	Expert system classification study	Dental disease classification	Hybrid fuzzy-AI / multilayer FIS + K-means	Multilayer fuzzy inference system with K-means centroiding	Classify dental diseases while reducing ambiguity between similar diseases	Symptoms for pulpitis, gingivitis, periodontitis, advanced periodontitis	21 expert training data; 15 test cases; 13 fuzzy rules (10 first layer, 3 second layer)	Expert prediction	Comparison of expert predictions and system classifications	15/15 tests matched expert predictions; accuracy 100%	Multilayer FIS + K-means reduced ambiguity and computational complexity, especially between pulpitis and gingivitis
Scrobotă et al.	2017	Original clinical/modeling study	Oral cancer risk assessment / OPMD screening	Fuzzy risk prediction / multicriteria DSS	MIN-MAX fuzzy logic; 25 IF-THEN inference rules; triangular/trapezoidal membership functions	Estimate oxidative-stress-related cancerization risk in oral potentially malignant disorders	Serum malondialdehyde (MDA) and proton donor capacity	16 patients with clinically/histopathologically diagnosed oral potentially malignant disorders	No external comparator; model outputs based on biochemical inputs	Fuzzy risk estimation on 1–10 scale	Risk generated as concrete numerical value; example high MDA/low proton donors produced risk 8.9813; system output range 1.656–9.344 in table	Potential aid for oral cancer screening and future decision-making in OPMDs
Scrobota et al.	2025	Original clinical/modeling study	Periodontitis risk assessment in type 2 diabetes	Fuzzy risk prediction / AI-based	Two-level fuzzy logic system: PRisk1 (BMI+glycemia), PRisk2 (cholesterol+tri	Estimate periodontitis risk in T2DM patients using accessible metabolic biomarkers	BMI, glycemia, total cholesterol, triglycerides; periodontal	87 T2DM patients selected from 300 dental-office patients	Clinical number of periodontal pockets (nrPP)	Regression/correlation between fuzzy PCRisk and nrPP	PCRisk correlated with nrPP; regression significant (p=0.021); nrPP = 0.399	Fuzzy system may support early periodontitis risk assessment and

				fuzzy DSS	glycerides), then PCRisk		pockets as clinical outcome				+ 1.881 × PCRisk	interdisciplinary diabetes-dental care
TinukeO & Yetunde	2015	Desktop expert system development	Dental ailment diagnosis	Neuro-fuzzy expert system	Coactive Neuro-Fuzzy Expert System (CANFES); C# desktop app; SQL database	Develop Electronic Dentist (eD) to diagnose mild dental problems from symptoms	Patient symptoms; binary symptom presence; fuzzy values; expert rules	No formal clinical sample; software demonstration	No formal comparator reported	System implementation/demonstration	Claims improved speed, efficiency, reliability vs manual approach; no quantitative diagnostic accuracy reported	Demonstrates practical neuro-fuzzy expert system architecture for dental diagnosis
Veryha & Adamczyk	2005	Fuzzy classification framework/ prototype	Primary dental care service assessment/patient feedback	Fuzzy classification framework	Fuzzy classification with membership functions; SQL-compatible relational database implementation	Assess patient feedback about primary dental care services by combining verbal and numeric criteria	Patient feedback criteria: appointment availability and waiting time; verbal/numeric survey data	Prototype/example dataset in Microsoft SQL Server	Conventional non-fuzzy classification	Prototype demonstration comparing conventional vs fuzzy classification	Fuzzy classification provided more accurate/descriptive evaluation of patient feedback and membership values for classes	Useful for quality assessment and practice management, not direct diagnosis/treatment
Wanti & Somantri	2022	Comparative expert-system classification study	Dental disease detection	Mamdani fuzzy logic expert system	Mamdani fuzzy logic compared with Naïve Bayes	Compare Mamdani fuzzy logic and Naïve Bayes for dental disease detection	Dental disease symptoms and supporting-tissue complaints from patient interviews/expert consultation	67 patients from North Cilacap community health centre, July–Dec 2021	Dentist/expert diagnoses	Confusion matrix vs expert diagnosis	Mamdani accuracy 85.1% vs Naïve Bayes 82.1%; Mamdani TP=46, TN=11, FP=8, FN=2	Mamdani fuzzy logic performed slightly better than Naïve Bayes for dental disease detection
