# Supplementary material for: Fuzzy Logic and Fuzzy-Based Artificial Intelligence in Dentistry: A Systematic Review
Source: Diagnostics (Basel). 2026 Aug 30;16(17):2787. doi: 10.3390/diagnostics16172787 (PMC13565610; doi:10.3390/diagnostics16172787)
Supplement: Supplementary file 1 [file diagnostics-16-02787-s001.zip › Supplementary Table S4.pdf]

Supplementary Table S4. Summary of the risk of bias outcomes

Study	Tool used	Overall judgment	Main reason
Ambara et al. (2017)	PROBAST	High	Expert-system model with limited/unclear independent validation
Adwibowo (2020)	JBI-style methodological appraisal	High	Conceptual/preprint fuzzy risk model; no clinical validation
Allahverdi & Akcan (2011)	PROBAST	High	Expert-rule system without external validation
Alsmadi (2018)	QUADAS-2	Some concerns	Manual reference used, but dataset selection/external validation unclear
Arna Fariza et al. (2018)	QUADAS-2	Some concerns	Real X-ray testing, but limited reporting of sampling/reference standard
Buhari & KotherMohideen (2020)	QUADAS-2	High	Unclear reference standard and validation procedures
Ismayilova & Tarverdiyeva (2023)	JBI-style methodological appraisal	High	Prototype expert system; limited empirical validation
Nasir et al. (2024)	PROBAST	High	Fuzzy expert system with example/testing only; no robust clinical validation
Parewe & Mahmudy (2016)	PROBAST	High	FIS model with unclear independent validation
Prandana et al. (2024)	PROBAST	High	Very small training/testing set; high overfitting risk despite 100% accuracy
Scrobotă et al. (2017)	PROBAST	High	Very small sample; risk score not externally validated against future cancerization
Scrobota et al. (2025)	PROBAST	High	Single-sample model development; no external validation despite clinical correlation
TinukeO & Yetunde (2015)	JBI-style methodological appraisal	High	Software/prototype paper without adequate diagnostic validation
Veryha & Adamczyk (2005)	JBI-style methodological appraisal	High	Fuzzy classification framework/prototype; no clinical outcome validation
Wanti & Somantri (2022)	QUADAS-2	Some concerns	Compared with expert diagnosis, but limited sample and unclear blinding

Note: For heuristic, rule-based, or primarily computational systems assessed using PROBAST, judgments were interpreted in the context of the study design. A high risk-of-bias judgment primarily reflects limitations relative to conventional prediction-model validation standards, such as small datasets, limited independent or external validation, or incomplete assessment against clinical reference standards, and should not be interpreted as indicating that the underlying computational methodology is inherently invalid.
